# Community-based management of chronic obstructive pulmonary disease in Nepal—Designing and implementing a training program for Female Community Health Volunteers

**DOI:** 10.1371/journal.pgph.0000253

**Published:** 2022-03-25

**Authors:** Tara Ballav Adhikari, Bishal Gyawali, Anupa Rijal, Abhishek Sapkota, Marieann Högman, Arjun Karki, Torben Sigsgaard, Dinesh Neupane, Per Kallestrup

**Affiliations:** 1 COBIN Project, Nepal Development Society, Bharatpur, Chitwan, Nepal; 2 Department of Public Health, Section for Global Health, Aarhus University, Aarhus, Denmark; 3 Global Health Section, Department of Public Health, University of Copenhagen, Copenhagen, Denmark; 4 Department of Internal Medicine, Holbæk Hospital, Holbæk, Denmark; 5 Department of Regional Health Research, The Faculty of Health Sciences, University of Southern Denmark, Odense, Denmark; 6 Department of Medical Sciences, Respiratory, Allergy and Sleep Research, Uppsala University, Uppsala, Sweden; 7 Department of Pulmonary, Critical Care and Sleep Medicine, HAMS Hospital, Kathmandu, Nepal; 8 Department of Public Health, Section for Environment, Occupation & Health, Aarhus University, Aarhus, Denmark; 9 Department of International Health, Johns Hopkins Bloomberg School of Public Health, Johns Hopkins University, Baltimore, Maryland, United States of America; Zuckerberg San Francisco General Hospital and Trauma Center, UNITED STATES

## Abstract

Chronic obstructive pulmonary disease (COPD) is one of the leading causes of morbidity and mortality in Nepal. Female community health volunteers (FCHVs) have proven effective in the delivery of reproductive, maternal, and child health services in Nepal and recently in the prevention and management of hypertension and type 2 diabetes. Evidence on their roles in COPD management is not yet available. The aim of this study was to develop, implement, and evaluate a training program for FCHVs regarding COPD prevention and management. The training program was part of a cluster-randomized trial of a 12-month intervention to improve COPD outcomes in a semi-urban area of Western Nepal. A six-day workshop consisting of thirty hours of training was developed for FCHVs. Training materials incorporated introduction to COPD, risk factors and symptoms, COPD status assessment guide for FCHVs, guidance on breathing techniques, and exercises for people living with COPD. Pre- and post-test questionnaires were administered to assess the change in knowledge of FCHVs, post training skills assessment followed by semi-structured interviews assessed FCHVs’ satisfaction with the training program. The findings of the pre- and post- test assessments showed a significant improvement in FCHVs’ COPD-related knowledge from a median (interquartile range) score of 12 (3–16) before to 21 (21–22) (p<0.001) after the training program. The qualitative assessment revealed the feasibility of FCHVs’ training on COPD and their acceptability to deliver the intervention package within the community. It also indicated that implementing future training with an extended period and a few days break in-between could enhance the effectiveness. Training of FCHVs in COPD management is feasible and leads to improvement in knowledge. The motivation shown by FCHVs to deliver the intervention could inform and guide community programs and policies for COPD prevention and management in Nepal and similar settings.

## Introduction

Globally, chronic obstructive pulmonary disease (COPD) has an increasing prevalence and constitutes a substantial socioeconomic burden [1]. In 2017, above 3% of disability-adjusted life years and 3.2 million deaths were ascribed to COPD worldwide [2]. A recently published meta-analysis estimated a 12% global prevalence of COPD [3]. More than 90% of COPD-related deaths occur in low-and middle-income countries (LMICs) [4]. COPD is the most prevalent non-communicable diseases (NCDs) in Nepal, with a 12% prevalence in adults [5]. In 2016, nearly one million Nepalese people were suffering from COPD, twice as many as in 1990 [6]. The higher burden of COPD in Nepal can be attributed to a high rate of tobacco smoking [7], substantial use of biomass fuel for cooking [8], poor outdoor air quality [9], exposure to second-hand smoking [8], and a demographic development with an aging population [10].

One of the major challenges in COPD prevention and management in Nepal’s already weakened health system, is the shortage of COPD specialists. The number of practicing pulmonologists in Nepal is relatively low, and most are based in the capital city Kathmandu or other big cities. Lack of proper diagnostics, treatment, and medications at peripheral health centers in combination with a low level of health literacy and awareness of the disease in the population constitute important challenges facing the increasing burden of COPD in Nepal [11–13]. COPD diagnosis is mostly limited to symptomatic assessment without objective confirmation by spirometry [12], potentially leading to underdiagnosis and misdiagnosis.

In a scenario with health workforce shortage, the shifting of tasks such as screening, education, referral, and follow-up to non-physicians such as community health workers (CHWs) can be an effective way to ensure the delivery of health care services [14]. The World Health Organization recommends optimizing community-based programs involving CHWs to prevent and manage NCDs, including COPD [15]. It is widely recognized that CHWs play an important role in a wide range of health behavior improvement and health outcome initiatives [16, 17]. Recently, CHW-led interventions have shown positive results in preventing and managing NCDs, including type 2 diabetes and hypertension [18]. Training programs for CHWs in monitoring and care of COPD patients in a rural part of India [19], and educational interventions for CHWs to increase awareness on COPD at a community level in Uganda [20] are some of the promising examples of CHWs’ engagement in community-based management of COPD in LMICs.

A network of CHWs, Female Community Health Volunteers (FCHVs), has been operating within the Nepalese health system for the last three decades [21]. FCHVs are local women selected by mothers’ groups in the communities receiving a short 18 days training. They serve as a bridge between the community and the health system, increasing health service utilization, promoting healthy behavior, and raising health awareness in the community [22]. Recent studies have shown promising results of the work of FCHVs in type 2 diabetes and hypertension management in Nepal [23, 24]. However, FCHVs’ involvement in COPD prevention and management has not yet been explored.

A research project entitled ’Community-based management of COPD in Nepal—a cluster-randomized controlled trial (COBIN-P)’ has been implemented in a collaboration between Aarhus University in Denmark and Nepal Development Society in a semi-urban area of Pokhara Metropolitan city of western Nepal [25]. This ongoing study aims to assess the effectiveness of an FCHV-led intervention to prevent COPD and improve disease management among adults with COPD. This study reports on the development and evaluation of the feasibility and preliminary effectiveness of a training program to strengthen and extend the knowledge of FCHVs in an intervention to prevent and manage COPD at community level.

## Methods

### Study setting and data collection

This study is part of the COBIN-P project. This two-arm cluster randomized controlled trial is ongoing in the semi-urban area of Pokhara Metropolitan city (former Lekhnath Municipality) of Western Nepal [25]. As a part of the study intervention, FCHVs were trained to raise awareness of prevention and management of COPD at the community level through household visits. FCHVs’ knowledge of COPD was assessed before and after the training program. Eight informants were recruited by convenience sampling to reach data saturation and eight in-depth interviews provided data on FCHVs’ feedback on the training program. The training program and data collection took place from December 2019 to February 2020. Similarly, prior to developing and designing the training program, eight stakeholders from the community (three FCHVs, two health assistants, one medical officer) and two health education experts were purposively selected.

### Developing a training program and educational materials for FCHVs

The study team developed the training program, including training curriculum and tools, based on an extensive literature review of similar CHWs-led interventions on the management of COPD and other NCDs. The team reviewed the health education materials previously used in tobacco and tuberculosis control, hypertension, and diabetes management programs in Nepal [26, 27]. The curriculum was developed by triangulating with domain experts, National Health Education and Information Center (NHEICC) professional staff, district health administrators, physicians, and FCHVs. As some of the community people and FCHVs were illiterate, locally appropriate tools were also developed, including pictorial flip charts and brochures for screening and management of COPD (S1 Appendix).

Based on a literature review, the training program was designed to provide FCHVs with knowledge regarding COPD risk factors, signs, and symptoms, management of COPD via screening, basic breathing techniques, stamina, endurance-building exercises, and knowledge about healthy lifestyle and medication adherence. Likewise, FCHVs were also trained to use educational materials during their home visits, and to identify and refer patients to the nearest health facility, and properly maintain recording and reporting forms. Every session was followed by skills-building sessions. Training was guided by a facilitator’s manual, ’Community Based Prevention and Management of COPD in Nepal: Female Community Health Volunteer’s Training Facilitator Guide’. The facilitators’ guide was developed to assist facilitators in being familiar with the module contents, session objectives, and delivery methods. The training materials, including the facilitator guide, were developed following procedures adopted for other COBIN studies on diabetes and hypertension [26, 27], and Engage-Tuberculosis Training of Community Health Workers and Community Volunteers: Facilitator’s Guide [28]. Experts were consulted for suggestions to be incorporated into the training program. Pilot testing of the training matrieals and program was performed, and adaptations were made. The FCHVs received training for 30 hours during a period of six days (S1 Table).

### Training of FCHVs

There were 123 FCHVs working in the study area. Fifty-seven FCHVs from the randomly assigned seven intervention clusters were invited for a one day orientation and assessment session. During the first day of training, FCHVs were informed about the COBIN-P project and introduced to COPD. FCHVs were then assessed for the minimum requirements of reading, writing, motivation, and availability to attend the next five days of training and the one-year intervention period. A total of 23 FCHVs fulfilled the requirements and were enrolled for the next five days of training. One FCHV dropped out of the training after two days due to personal reasons. The training program was delivered by the principal investigator (TBA), a medical officer from the Ministry of Health and Population who was also a certified trainer of World Health Organization package of essential NCDs, a consultant pulmonologist, and two health staffs from the local organization Nepal Development Society with previous experience in NCDs-related training. The training sessions were conducted in the local language (Nepali) using PowerPoint presentations, videos, demonstrations, and group exercises. The six-day FCHVs training package consisted of seven units and 16 lessons summarized in S1 Table.

### Evaluation

The training program was evaluated using both quantitative and qualitative methods. FCHVs completed a short questionnaire in the local language measuring their degree of COPD knowledge before and after training. The knowledge assessment questionnaire (S2 Appendix and S3 Appendix) was adapted from previous studies [20, 29]. Participants completed the questionnaire in strict confidentiality. Similarly, the skills of FCHVs were assessed on teaching breathing exercises, stamina and endurance building exercise, skills on using a flip chart, and COPD status assessment guide during and at the last day of the training using a skill assessment evaluation form. All FCHVs performed the required tasks during the assessments. Also, the field supervisor regularly monitored and supervised the FCHVs throughout the phase of trial implementation using an evaluation and supervision form (S4 Appendix). We conducted in-depth interviews with three FCHVs, two health assistants and one medical doctor, at the primary health care centers in the study area while designing the training package and educational material for the intervention. Through these pre-training interviews, we explored the health-seeking behavior for COPD, provision of health service delivery for COPD, and the importance and components of the community-based program to prevent and manage COPD. One month after the training, eight FCHVs were interviewed to assess their experiences with the training, obstacles, and challenges in delivering the intervention in the community using topic guide for the interview (S5 Appendix and S6 Appendix). Two trained researchers (TBA and AS) with a degree in public health and previous experiences in facilitating in-depth interviews conducted the interviews.

### Data analysis

Demographic characteristics of FCHVs were summarized using means and percentages. Each knowledge statement was recorded as a dummy variable, scoring 1 for correct and 0 for incorrect responses. The proportion of participants with correct responses for every statement on the questionnaires was determined and compared before and after using the McNemar test. The overall score was calculated by adding the dummy score 1 and 0 before and after the training. The overall change in knowledge score before and after the training was assessed using the Wilcoxon signed-rank test. All statistical tests were two-sided at alpha = 0.05. Data were entered in Epi Data version 4.0 (The EpiData Association, Odense, Denmark) and analyzed using STATA version 15.1 (StataCorp. Texas, USA). Data are presented as mean ± standard deviation (SD). FCHVs providing verbal consent and permission to audio-record the interviews were included in the qualitative study. The interviews were audio-recorded in the local language, transcribed verbatim and later translated into English. A thematic analysis was conducted for transcripts from interviews [30]. It included the steps of iterative reading of interview transcripts, creating initial codes, arranging the codes, and generating a thematic map of the analysis. Two researchers (TBA and AS) analyzed the qualitative data independently, and consensus regarding the nature and coding of emerging themes was reached through discussion with other team members.

#### Ethics approval and consent to participate

The study was conducted in accordance with the Declaration of Helsinki. Ethical approval was obtained from the review board of the Nepal Health Research Council (Approval number: 30–2019), and written informed consent was obtained from all participants.

## Results

### Quantitative findings

Twenty-two FCHVs participated in this study with a mean age of 45 ± 8 years. The majority belonged to an advantaged ethnic community with average schooling years of 9 ± 2 years. The mean years of working as an FCHV was 17 ± 10 years.

Six FCHVs had never heard of COPD before and more than 40% were not aware that it was a lung disease. Only three FCHVs were aware of the breathing test for COPD diagnosis and the importance of vaccination for pneumonia and influenza. Likewise, none of the FCHVs had knowledge of and skills in breathing exercises and techniques for people living with COPD. Prior to training, the median (interquartile range) correct COPD-related knowledge score of FCHVs was 12 (3–16); after training this increased to 21 (21–22) (p<0.001) (Table 1).

**Table 1:** Results of Female Community Health Volunteers’ COPD-related knowledge before and after training (n = 22).

COPD knowledge statements	Correct in pre-test	Correct in post-test	p-value
	Number (%)	Number (%)	
Heard about COPD	16 (73)	22 (100)	0.03†
COPD is a disease of the Lung.	13 (59)	22 (100)	0.04†
COPD is most prevalent NCD in Nepal	14 (64)	20 (91)	<0.001†
COPD is a chronic condition	13 (59)	22 (100)	<0.001†
COPD is a preventable disease	16 (73)	21 (96)	<0.001†
COPD is unusual in people below the age of 40 years	17 (77)	22 (100)	<0.001†
In COPD, there is usually gradual worsening over time	12 (55)	22 (100)	0.002†
Breathing tests confirm COPD	3 (14)	18 (82)	<0.001†
In COPD, oxygen levels in the blood are always low	4 (18)	20 (91)	<0.001†
**Knowledge on symptoms of COPD**			
Cough	10 (46)	22 (100)	<0.001†
Phlegm production	13 (59)	22 (100)	0.001†
Shortness of Breath	13 (59)	22 (100)	0.004†
Wheezing	7 (32)	22 (100)	<0.001†
Don’t know any symptoms	7 (32)	0 (0)	0.02†
**Knowledge of risk factors of COPD**			
Tobacco smoking	11 (50)	22 (100)	0.001†
Biomass fuel smoke	12 (55)	22 (100)	0.002†
Outdoor air pollution	8 (36)	21 (96)	<0.001†
Alcoholism	17 (77)	22 (100)	0.06†
Don’t know any risk factors	6 (27)	0 (0)	0.03†
**Knowledge of biomass fuels, smoking and COPD**			
Stopping smoking will keep COPD from getting worse	13 (59)	22 (100)	0.004†
Avoiding biomass fuel smoke prevents disease from getting worse	5 (23)	22 (100)	<0.001†
Cigarette smoking or second-hand smoke causes most cases of COPD	9 (41)	22 (100)	<0.001†
**Knowledge of treatment and medication of COPD**			
People with COPD should get vaccinated against influenza and pneumonia	3 (14)	22 (100)	<0.001†
COPD medicines (inhalers) prevent the disease from getting worse	15 (68)	22 (100)	0.02†
Knows about breathing exercise and techniques for COPD	0 (22)	21 (96)	<0.001†
Walking and physical activity helps to improve fitness and lung health	7 (32)	20 (91)	<0.001†
Overall Median Score (Inetrquartile range)	12 (3–16)	21 (21–22)	<0.001††

†McNemar test

††Wilcoxon signed-rank test.

### Qualitative findings

#### Stakeholder interviews before designing the training materials

Consultative meetings and interviews with eight concerned stakeholders from the community (three FCHVs, two health assistants, one medical officer) and health education experts (two public health officers from NHEICC) were conducted before designing the training package. All interviews highlighted the importance of the FCHV training on COPD management, particularly the improvement of the knowledge of COPD and its risk factors. FCHVs reported that training would enhance early consultations of symptomatic cases in health centers.

*"In our village, using firewood for cooking is common. (However, there is) limited knowledge regarding its long-term health effects." (FCHV1)*

*"We (FCHVs) can provide education on using of the smokeless cooking stove, motivate establishment of well-ventilated kitchens if (there is) no possibility of smokeless stoves, (remain) away from smokes of vehicles and suggest not to burn plastics (waste)." (FCHV2)*

*"People ignore COPD signs and symptoms as normal cough, aging issue, and results of seasonal changes, thus resulting in adverse health effects. Therefore, FCHVs can provide health messages in the community and refer individuals to us (health centers) as early as possible." (Medical Officer)*

#### Challenges in COPD prevention and management

Two kinds of barriers in COPD prevention and management in the community were identified, including the lack of knowledge and awareness regarding the disease and insufficient services related to COPD at the health centers.

*"There is no adequate treatment of COPD and no medicines available in our health posts in this village. That is why they (people) do not visit the local health facility." (FCHV 3)*

*"For the proper treatment, firstly patient should visit the health posts as early as possible so that we can check them with our capacity and, if not refer to hospitals. But very few come to us due to lack of awareness about this disease." (Health assistant)*

Similarly, the degrading outdoor air quality was also considered a challenge in COPD prevention and management. One of the FCHVs said: *"We can request them to make their kitchen smokeless based on their capacity*. *How can we protect the dust of roads which are under construction*?*" (FCHV 1)*

#### Increased knowledge of and skills in COPD after the training

FCHVs reported that the training enhanced their knowledge regarding COPD. One FCHV stated: "*I was not aware of other lung health problems other than pneumonia*, *(lung) cancer and asthma*. *Now*, *I can share information with community (people) about COPD and ways to prevent it*.*" (FCHV 4)*

The FCHVs acknowledged that training enhanced their skills to assess COPD status with simple questions and that made them able to help people living with COPD in their community.

"*I used to see many people with (respiratory) symptoms and was unable to help them*. *Now*, *I can assess with (CAF) guide*, *make suggestions*, *and refer them if health (condition) is poor*. *People (with COPD) are happy when I teach them breathing techniques and (endurance and stamina building) exercise*.*" (FCHV 7)*

"Along with regular things, having diabetes and hypertension management skills had enriched respects in community. In addition, now we can teach people about new (COPD) disease." (FCHV 6)

#### Satisfaction with the training package

The trained FCHVs were pleased with the training package and the way it was delivered methodology. The diversity in trainers from senior pulmonologists to local health workers was also well received by the FCHVs.

*"(I am) happy with this training. I gained lots of knowledge through different (trainers) people in COPD. And also (we) had the opportunity to practice and get feedback on making mistakes." (FCHV 5)*

However, some mentioned time limitations in training considering the novelty of COPD to participants. *"Being a new disease and different kind of information*, *(I) had a difficult time to catch (grasp) everything*. *Few more days of training with breaks for a few days could be (better)*.*" (FCHV 9)*

#### Possible challenges to the implementation of the program

FCHVs considered that the community people were receptive to their work. People living with COPD were very responsive during home visits. However, they underlined a few challenges, mainly among people without the disease who expressed less interest in hearing about treatment.

*"We have respect in the community, so they do not directly reject us. I found that people with the disease (COPD) were responsive to our home visits. They felt good about exercise. But some people without disease were less interested in hearing about quitting smoking and treatments." (FCHV 11)*

One of the FCHVs highlighted financial and health system challenges for the implementation: *"Ok*, *I referred some of them to health posts*, *and if they do not find proper service and treatment there*, *then they do not want us to hear in next (home) visit*. *Many poor people cannot go to Kathmandu (tertiary hospitals)*. *Then I have to be quiet despite (poor) health when I go (visit) them"*. *(FCHV 5)*

Likewise, FCHVs demanded extra incentives for additional roles. *"We are providing new services to the community*, *but incentives are almost unchanged*. *Therefore*, *with work related to a new disease*, *we wish for extra monetary support for us*.*" (FHCV 8)*

#### Suggestions for future improvement of training

FCHVs regarded the training as very important to curb the rising burden of COPD in the community, with few suggestions on an integrated program for NCDs. They highlighted the need for refresher training on a timely basis.

*"When I visit for this (COPD) or diabetes (another project in study site), I find other NCDs in a particular household. So, now it would be better for us if you train us for combined (integrated) work." (FCHV 10).*

*"Refresher training every 5–6 months is better. So that we can update (on knowledge and skills). We are not that educated and growing older so we may forget many things. I request the Nepal Development Society (local implementing organization) to provide training regularly." (FCHV 6)*

## Discussion

In this study, we developed, implemented, and evaluated a first-of its kind, COPD prevention and management training program for FCHVs in Nepal. We found that following participation in this structured training program, FCHVs’ knowledge of COPD significantly (p<0.001) increased from a median knowledge score of 12 to 21 (total score = 24). Before the training, a relatively low number of FCHVs knew that smoking causes COPD and that avoiding biomass fuel smoke prevents the disease from worsening. Similarly, only three out of 22 FCHVs knew the importance of influenza and pneumonia vaccinations for patients with COPD. Overall, we observed a knowledge insufficiency in the fundamental domains of symptoms, risk factors, and treatment of COPD among FCHVs, which significantly improved after our training program.

One of the strengths of this FCHVs training was that the training was based on a comprehensive literature review, stakeholder meetings, utilization of existing nationally endorsed health promotion messages, and tailored to the community of the study area. The training was delivered through presentations, videos, role plays, demonstrations, and group exercises, as recommended by previous studies [19, 31]. All these elements increased the effectiveness of the training. We believe this training package with necessary community-specific modifications can be replicated in similar settings in other parts of Nepal. It must be considered that some FCHVs found that continuous training for six days was challenging to participants to comprehend all the new knowledge at once, although we incorporated fun games, local singing, and dancing in between sessions to engage participants and to give a sense of break so that the training was not overwhelming. The qualitative assessment of FCHVs showed motivation and interest among FCHVs in delivering the intervention in the community, and FCHVs perceived that the community was responsive. FCHVs became confident in using the status assessment guide and making referrals. In the context of Nepal, where the objective diagnosis of COPD in primary healthcare centers is very low, and COPD is largely undiagnosed in the community [12, 32], FCHVs can be trained in using short screening questionnaires to screen potential COPD cases in the community. Several short and simple COPD screening tools are being used elsewhere [33], which could be validated in Nepal for community-based screening of COPD by FCHVs.

A noteworthy finding from the qualitative assessment indicated that community-based COPD prevention and management would be challenging to implement and less effective unless local health facilities are strengthened in terms of diagnosis, management, and treatment. Therefore, along with community-based interventions, efforts should be made to strengthen the local health facility’s capacity to effectively diagnose and treat COPD at an affordable cost. So those potential COPD patients referred by CHWs from the community who otherwise would have been missed are identified early and treated appropriately. Also the strengthening of the health system’s readiness and response to CHWs referrals with proper diagnosis, treatment, and medication is needed to increase the trust and effectiveness of CHWs in the community [34].

Patient activation and empowerment for self-management are essential in managing COPD and improving quality of life [35]. In our study, FCHVs were confident in delivering home-based counseling on COPD and demonstrating breathing techniques, stamina and endurance building exercises, support on symptoms assessment, and bridging the gap to health services. A feasibility study conducted in the UK showed lay health workers being acceptable and supportive in uptake and completion of pulmonary rehabilitation in COPD [36]. This sheds light on the need for future research on FCHVs involvement in pulmonary rehabilitation for COPD in resource-limited settings like Nepal.

The widely discussed remuneration and overburdening of CHWs with multiple roles is a matter of concern and also revealed in our qualitative findings [34, 37]. The remuneration of CHWs is considered an essential factor for motivation [37]. In our research, we provided transportation costs as remuneration for their work following the government guidelines of FCHVs incentives. FCHVs also suggested that integrated training, i.e., training on diabetes, hypertension, and COPD could be important as number of households with multiple NCDs are increasing in the community. The integrated package of NCDs for CHWs routine work would eventually reduce the burden and empower FCHVs to help community people comprehensively, but further research and policy discourses on this aspect are needed.

This study presented the development and evaluation of a training program for implementing an FCHV-led model of COPD prevention and management in a community in Nepal. This study had some limitations. The relatively small sample size of trained FCHVs and conveniently selected participants could limit the generalizability of our findings. FCHVs received our training program well; however, this research was implemented by an organization with previous experience in implementing FCHV-led interventions in type 2 diabetes and hypertension management at the same study site [23, 38]. Therefore, FCHVs may have overstated the effect because they do not want to be impolite to the research team. Nevertheless, when triangulating with a knowledge questionnaire, our findings were confirmed. Also, we cannot exclude that FCHVs might face some challenges in counseling some male participants in the context of patriarchal societal norms. However, noting the three-decade well-accepted presence and experience of FCHVs working in the community might partially address this issue. Other COBIN interventions were also implemented mobilizing FCHVs, and they did not face this challenge. We expect there would not be cultural issues that FCHVs may face in their interaction with male COPD patients [23, 38]. Likewise, counseling is considered an important component of this training program, and FCHVs are well-trained in culturally appropriate counseling. Finally, the COPD knowledge questionnaire in this study was adapted from previous studies conducted in Uganda and Turkey [20, 29]; therefore, contextualization and validation of the COPD knowledge assessment questionnaire in Nepal seems necessary for future studies.

The current COPD-related national program and policies in Nepal do not address COPD in the community and do not provide adequate directions to minimize the potential public health impact [39]. Thus, despite these limitations, this study as the first of its kind in Nepal to develop a training program for FCHVs to prevent and manage COPD, opens a discourse for further research in mobilizing local health resources to strengthen community health system to combat COPD.

## Conclusions

The findings of this study suggest that a short training program for FCHVs in COPD management is feasible in a semi-urban area of Nepal and improves knowledge significantly. We found that FCHVs were motivated and wished to expand their knowledge of COPD. This approach could be a promising model to mobilize health care resources and skills to address the increasing burden of COPD and its risk factors in Nepal and similar settings.

## Supporting information

S1 Table Summary of COPD prevention and management training for FCHVs in Nepal. (DOCX)

S1 Appendix COBIN P flip chart used by FCHVs. (DOCX)

S2 Appendix COBIN-P COPD knowledge assessment questionnaire (English). (DOCX)

S3 Appendix COBIN-P COPD knowledge assessment questionnaire (Nepali). (DOCX)

S4 Appendix COBIN-P FCHVs knowledge and skills evaluation/supervision form. (DOCX)

S5 Appendix Topic guide for COBIN-P key informant in-depth interview for FCHVs, and health workers (English). (DOCX)

S6 Appendix Topic guide for COBIN-P key informant in-depth interview for FCHVs, and health workers (Nepali). (DOCX)
