# Supplementary material for: Community-based management of chronic obstructive pulmonary disease in Nepal—Designing and implementing a training program for Female Community Health Volunteers
Source: PLOS Glob Public Health. 2022 Mar 25;2(3):e0000253. doi: 10.1371/journal.pgph.0000253 (PMC10021247; doi:10.1371/journal.pgph.0000253)
Supplement: S1 Table — (DOCX) [file pgph.0000253.s001.docx]

**S1 Table: Summary of COPD prevention and management training for Female Community Health Volunteers, Nepal**

| **Unit** | **Lesson** | **Topic** | **Contents** | **Materials and Methods** | **Duration** |
| --- | --- | --- | --- | --- | --- |
| **1** | Lesson 1 | COBIN-P project introduction, overviews of NCDs in Nepal | COBIN-P project and its objective, breif introduction of NCDs in Nepal. | **Materials:** FCHV training manual, facilitator guide, marker and white board. **Methods:** Powerpoint presentations and discussion. **Evaluation**: Questionaire and interview. | 60 min |
| **2** | Lesson 2 | Introduction of COPD and its situation in Nepal | Introduction to chronic respiratory disease, including COPD and situation of COPD in Nepal. | **Materials:** FCHV training manual, facilitator guide, model trachea, marker and white board. **Methods:** Powerpoint presentations and discussion. **Evaluation:** Questionaire and interview. | 60 min |
|  | Lesson 3 | Risk factors/causes of COPD | Tobacco smoking, indoor air pollution, occupational exposures, outdoor air pollution, lung growth and development, asthma and airway hyper-reactivity, genetic factors, age and sex. | **Materials:** FCHV training manual, facilitator guide, newsprint, marker, white board, metacard and types of ciggartaes. **Methods:** Demonstration, presentation, discussion and teach back. **Evaluation**: Questionaire and interview. | 60 min |
|  | Lesson 4 | Symptoms of COPD | Dyspnea/shortness of breath, chronic cough, sputum production, wheezing and chest tightness, additional features in severe disease like fever, infections. | **Materials:** FCHV training manual, facilitator guide, model trachea marker and white board. **Methods:** Powerpoint presentations, discussion and teach back. **Evaluation**: Questionaire and interview. | 60 min |
| **3** | Lesson 5 | Diagnosis and treatment of COPD | Spirometry, smoking cessation, avoiding exposure to dust, dirt, chemicals, and gases can irritate and inflame the airways and lungs, physical activity, vaccination, pulmonary rehabilitation can improve well-being. Medicines for COPD treatment:   1. Bronchodilators (inhalers) to open the airways 2. Steroids to reduce airway inflammation 3. Antibiotics to treat respiratory infection | **Materials:** FCHV training manual, facilitator guide, model trachea, Spirometer, bronchodilators, inhalers types, salbutamol tablets, marker and white board. **Methods**: Powerpoint presentations, discussion and teach back. **Evaluation:** Questionaire and interview | 60 min |
|  | Lesson 6 | Prevention and control of COPD | Ways of prevention and control of COPD 1. Quit Smoking, 2. Avoid secondhand smoking, 3. Improved indoor air quality, 4. Avoid outdoor air pollution 5. Balanced diet 6. Physical activity | **Materials:** FCHV training manual, facilitator guide, newsprint, marker, white board, metacard and types of ciggartaes. **Methods:** Demonstration, presentation and discussion and teach back. **Evaluation:** Questionaire and interview. | 60 min |
| **4** | Lesson 7 | COPD status assessment guide for FCHVs (CAF guide) | 1. Introduction to CAF guide: To assess the status of COPD using COPD status assessment guide for FCHVs (CAF guide) 2. Make recommendations accordingly based on disease status using CAF guide. | **Materials:** FCHV training manual, facilitator guide, newsprint, marker, white board, metacard and CAF guide. **Methods:** Demonstration, presentation and discussion. **Evaluation:** Questionaire and interview | 120 min |
|  | Lesson 8 | Practical session for using CAF guide | Know the skills to use the CAF guide and make recommendation. | **Materials:** FCHV training manual, facilitator guide, newsprint, marker, white board, metacard and CAF guide. **Methods:** Role play, hands-on-practice and discussion. **Evaluation:** Questionaire, observation and individual assessment. | 180 min |
| **5** | Lesson 9 | Breathing techniques and stamina and endurance building exercise for people living with COPD | 1. Teach the breathing techniques for COPD patients and people with Symptoms of COPD 2. Teach stamina and endurance building exercise for COPD patients and people with Symptoms of COPD | **Materials**: FCHV training manual, facilitator guide, newsprint, marker, white board, metacard and breathing techniques and exercise guide. **Methods:** Demonstration, presentation, and discussion. **Evaluation:** Questionaire and interview | 120 min |
|  | Lesson 10 | Practical session for breathing techniques and stamina and endurance building exercise for people living with COPD | Know the skills to teach breathing techniques and stamina and endurance building exercise for people living with COPD. | **Materials:** FCHV training manual, facilitator guide, newsprint, marker, chair white board, metacard and breathing techniques and exercise guide. **Methods:** Role play, hands-on practice, group work, teach back and discussion. **Evaluation:** Questionaire and observation. | 180 min |
| **6** | Lesson 11 | Counselling and health promotion messages on disease and risk factors using health education materials | Know the counseling techniques; provide health education for reducing modifiable risk factors using flip charts and brouchers. | **Materials:** FCHV training manual, facilitator’s guide, flip chart, brouchr, CAF guide, excercise guide, flash card, white board, marker and newsprint. **Methods:** Demonstration, presentation, discussion and role play. **Evaluation:** Observation Questionaire and interview | 120 min |
|  | Lesson 12 | Practical session for counselling | Know the practical way of counseling techniques; provide health education practically. | **Materials**: FCHV training manual, facilitator’s guide, flip chart, broucher, CAF guide, excercise guide, flash card, white board, marker and newsprint. **Methods**: Role play, hands-on practice and discussion. **Evaluation**: Questionaire, interview and individual assessment. | 180 min |
| **7** | Lesson 13 | FCHVs home visits | Preparation for follow up; when to make the first visit; when to follow up; how to visit the participants households. | **Materials**: FCHV manual, facilitator’s guide, FCHV register, follow up time schedule, calendar and referral form. **Methods:** Powerpoint presentation, role play and individual practice. **Evaluation:** Questionaire, interview and individual assessment. | 180 min |
|  | Lesson 14 | Recording and reporting | Know the detailed format of record, report, record correctly after home visit. | **Materials:** FCHV training manual, facilitator’s guide, FCHV register, record form, pencil and eraser. **Methods:** Presentation hands-on practice, teach back and discussion. **Evaluation:** Questionaire, interview and induvial assessment | 180 min |
| **8** | Lesson 15 | Selection of household | Make a list of households that FCHVs have to visit. | **Materials**: FCHV training manual, facilitator’s guide, pen, pencil, list of ward wise respondents with address and contact number. **Methods:** Group discussion. **Evaluation:** Questionaire and interview | 60 min |
|  | Lesson 16 | Evaluation and certification | Visit respondents’ household, provide counseling on reducing risk factors and record and maintain the FCHV register, CAF guide use, Breathing techniques and excericise skills. | **Materials:** FCHV training manual, facilitator’s guide, FCHV register, notebook, ballpoint pen, name tag, pencil, sharpener, eraser, shoulder bag, feedback form, CAF guide, flip chart and brouchers. **Methods:** Discussion and questions | 120 min |
