# Supplementary material for: Community-based management of chronic obstructive pulmonary disease in Nepal—Designing and implementing a training program for Female Community Health Volunteers
Source: PLOS Glob Public Health. 2022 Mar 25;2(3):e0000253. doi: 10.1371/journal.pgph.0000253 (PMC10021247; doi:10.1371/journal.pgph.0000253)
Supplement: S1 Appendix — (DOCX) [file pgph.0000253.s002.docx]

**Supporting Information**

**S1Appendix: COBIN P Flip Chart used by FCHVs**

**
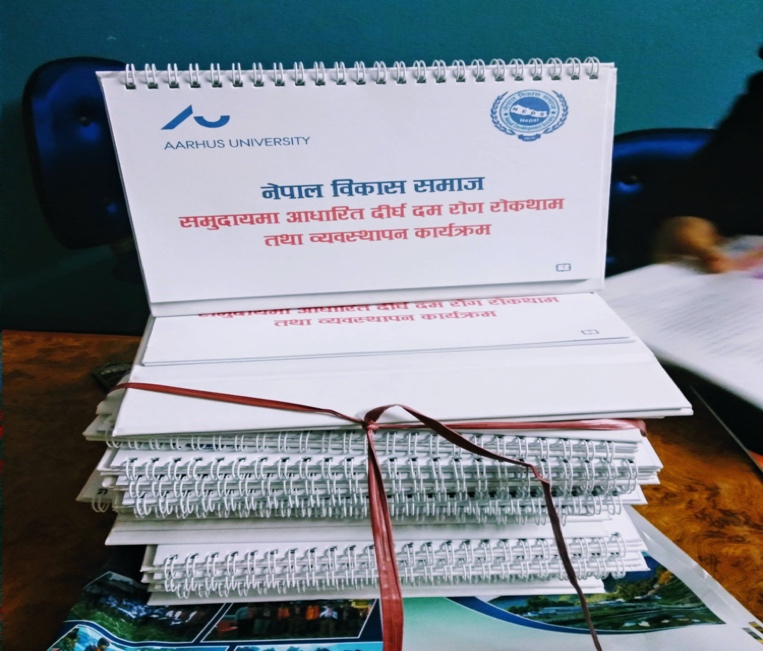
**

Pictures of healthy and COPD lungs

**
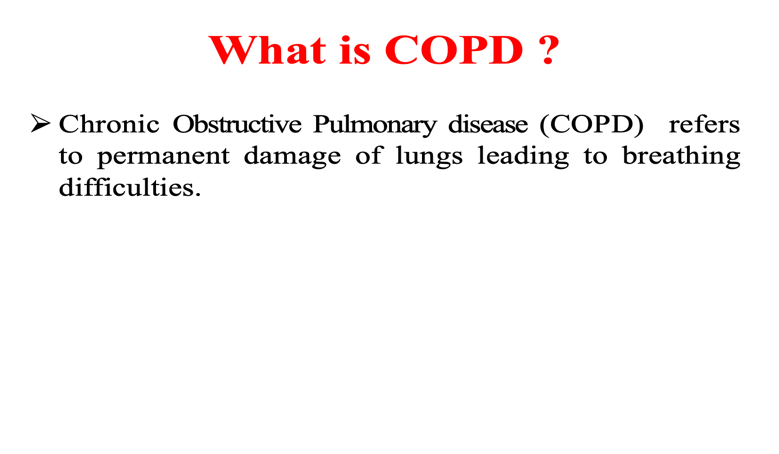
**

**
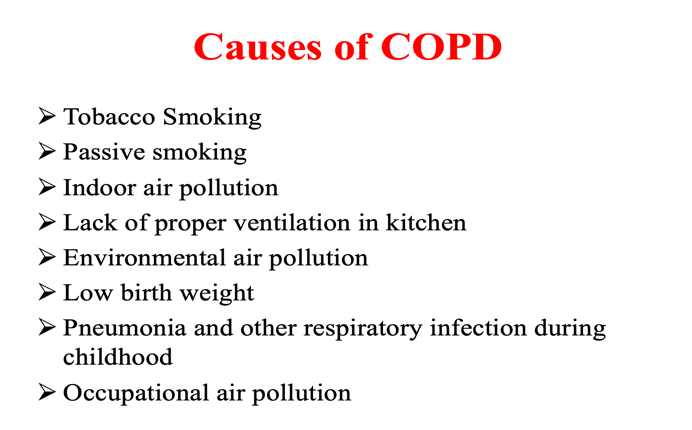
**

Figures demonstrating the causes of COPD

**
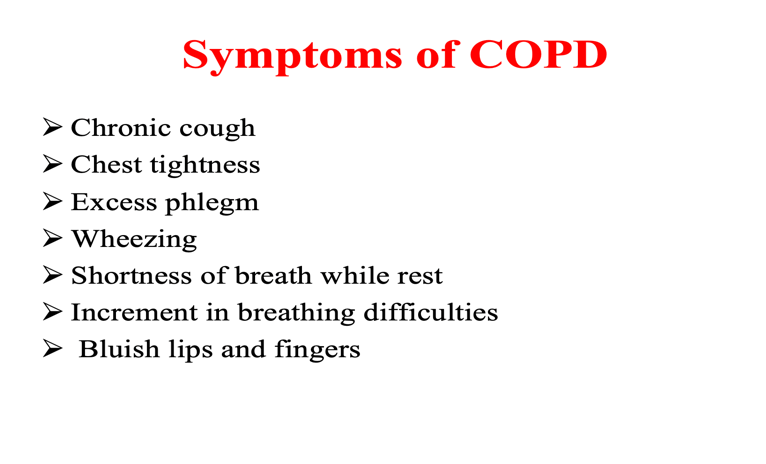
**

Pictures of Patient coughing and facing difficulty in breathing.

Pictures of COPD diagnosis by spirometry, chest x ray and blood test

**
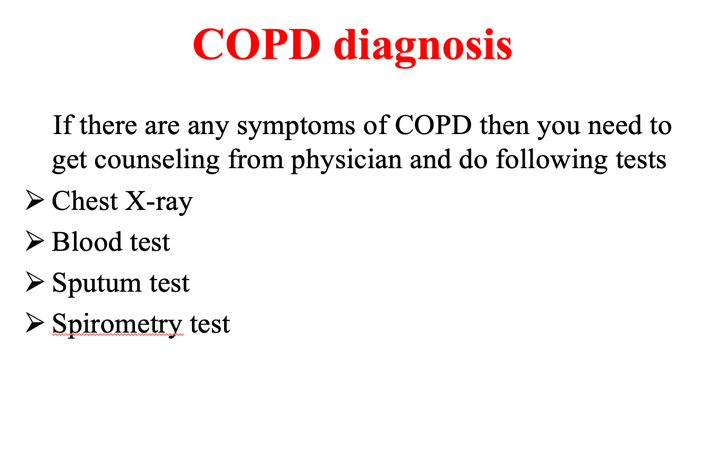
**

**
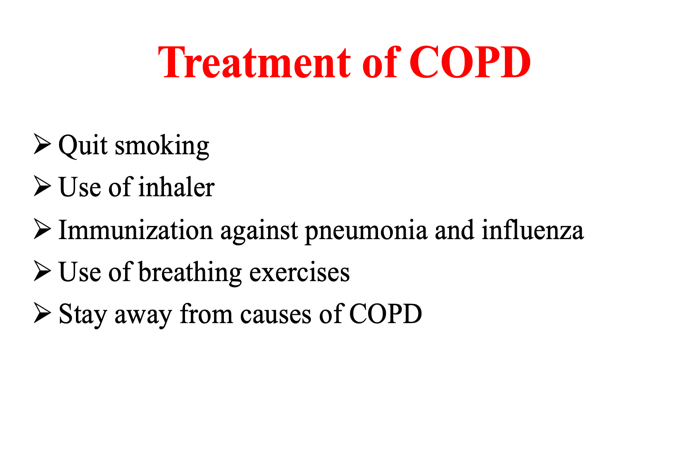
**

Pictures of COPD treatment demonstrating use of inhaler, vaccination, breathing exercises and quitting smoking.

**
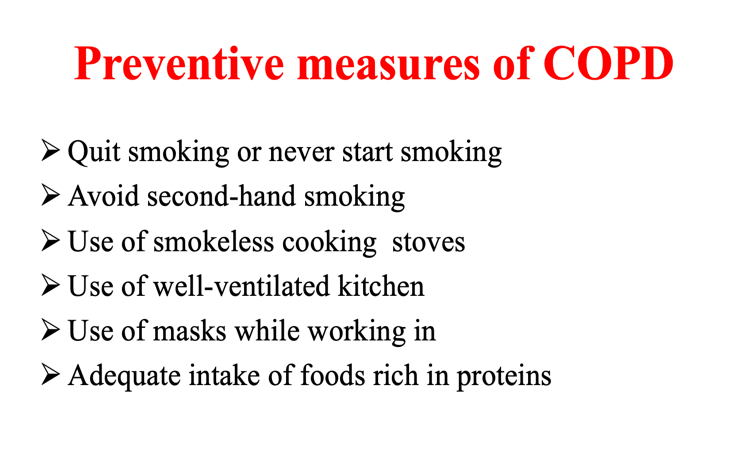
**

Pictures demonstrating well ventilated kitchen, smokeless cooking stoves, balanced diet, and avoiding/quitting smoking.

**
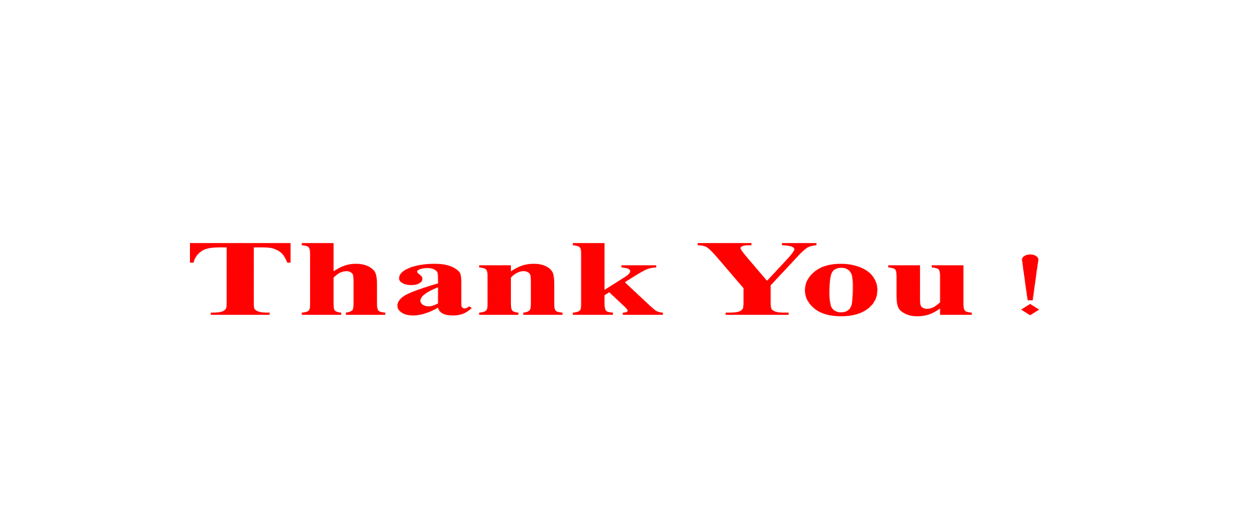
**
