# Supplementary material for: Community-based management of chronic obstructive pulmonary disease in Nepal—Designing and implementing a training program for Female Community Health Volunteers
Source: PLOS Glob Public Health. 2022 Mar 25;2(3):e0000253. doi: 10.1371/journal.pgph.0000253 (PMC10021247; doi:10.1371/journal.pgph.0000253)
Supplement: S2 Appendix — (DOCX) [file pgph.0000253.s003.docx]

**S2_Appendix: COBIN-P COPD Knowledge Assesment Questionnaire (English)**

Name: Address: Contact number:

Date of birth: Age: Health center:

Completed education level: Date starting to work as FCHV:

|  | **Knowledge statements** | **Response** | | |
| --- | --- | --- | --- | --- |
| 1 | Have you heard about COPD? | Yes | No | Don’t Know |
| 2 | Is COPD a lung disease? | Yes | No | Don’t Know |
| 3 | COPD is most prevalent NCD in Nepal. | Yes | No | Don’t Know |
| 4 | Is COPD a chronic condition? | Yes | No | Don’t Know |
| 5 | Is COPD a preventable disease? | Yes | No | Don’t Know |
| 6 | COPD is unusual in people below the age of 40 years? | Yes | No | Don’t Know |
| 7 | In COPD, there is usually gradual worsening over time? | Yes | No | Don’t Know |
| 8 | Breathing tests confirm COPD. | Yes | No | Don’t Know |
| 9 | In COPD, oxygen levels in the blood are always low? | Yes | No | Don’t Know |
| **What are the symptoms of COPD?** | |  |  |  |
| 10 | Cough | Yes | No | Don’t Know |
| 11 | Phlegm production | Yes | No | Don’t Know |
| 12 | Shortness of Breath | Yes | No | Don’t Know |
| 13 | Wheezing | Yes | No | Don’t Know |
| 14 | Don’t know any symptoms | Yes | No | Don’t Know |
| **What are the risk factors of COPD** | |  |  |  |
| 15 | Tobacco smoking | Yes | No | Don’t Know |
| 16 | Biomass fuel smoke | Yes | No | Don’t Know |
| 17 | Outdoor air pollution | Yes | No | Don’t Know |
| 18 | Alcoholism | Yes | No | Don’t Know |
| 19 | Don’t know any risk factors | Yes | No | Don’t Know |
| **Knowledge of biomass fuels, smoking and COPD** | |  |  |  |
| 20 | Stopping smoking will keep COPD from getting worse? | Yes | No | Don’t Know |
| 21 | Avoiding biomass fuel smoke prevents disease from getting worse? | Yes | No | Don’t Know |
| 22 | Cigarette smoking or second-hand smoke causes most cases of COPD? | Yes | No | Don’t Know |
| **Knowledge of treatment and medication of COPD** | |  |  |  |
| 23 | People with COPD should get vaccinated against influenza and pneumonia? | Yes | No | Don’t Know |
| 24 | COPD medicines (inhalers) prevent the disease from getting worse? | Yes | No | Don’t Know |
| 25 | Knows about breathing exercise and techniques for COPD? | Yes | No | Don’t Know |
| 26 | Walking and physical activity helps to improve fitness and lung health? | Yes | No | Don’t Know |
