# Supplementary material for: Community-based management of chronic obstructive pulmonary disease in Nepal—Designing and implementing a training program for Female Community Health Volunteers
Source: PLOS Glob Public Health. 2022 Mar 25;2(3):e0000253. doi: 10.1371/journal.pgph.0000253 (PMC10021247; doi:10.1371/journal.pgph.0000253)
Supplement: S3 Appendix — (DOCX) [file pgph.0000253.s004.docx]

**S3_Appendix:** COBIN-P COPD Knowledge Assessment Questionnaire (Nepali)

**दिर्घ दम रोग को ज्ञान र अवधारणा बारे प्रश्न हरु**

**नाम: ठेगाना: सम्पर्क नम्बर:**

**जन्म मिति: उमेर: स्वास्थ्य केन्द्र:**

**पूरा भएको शिक्षा स्तर: FCHV को रूपमा काम सुरु गर्ने मिति:**

|  | **k\|Zgx?** | **hjfkmx?** | | |
| --- | --- | --- | --- | --- |
| 1 | के तपाईंले दिर्घ दम रोगको बारेमा सुन्नु भएको छ ? | छ | हैन | थाहा छैन |
| 2 | के दिर्घ दम फोक्सोको रोग हो ? | हो | हैन | थाहा छैन |
| 3 | दिर्घ दम नेपालमा धेरै संख्यामा पाइने नसर्ने रोग हो । | हो | हैन | थाहा छैन |
| 4 | के दिर्घ दम एक दिर्घ समस्या हो? | हो | हैन | थाहा छैन |
| 5 | के दिर्घ दम रोगको रोकथाम गर्न सकिने रोग हो? | हो | हैन | थाहा छैन |
| 6 | ४० बर्ष भन्दा कम उमेरका मानिसहरुलाई सामान्यतय दिर्घ दम रोग हुँदैन ? | हो | हैन | थाहा छैन |
| 7 | दिर्घ दम रोगको समस्या समय संग बिस्तारै बिग्रिदै बल्झिदै जान्छ । | हो | हैन | थाहा छैन |
| 8 | दिर्घ दम रोगको निदान श्वास प्रश्वासको जांच बाट गर्न सकिन्छ . | हो | हैन | थाहा छैन |
| 9 | दिर्घ दम रोग हुँदा रगत मा अक्सिजन को मात्रा काम हुन्छ । | हो | हैन | थाहा छैन |
| **दिर्घ दम रोग का लक्षणहरू बारे** | |  |  |  |
| 10 | खोकी | हो | हैन | थाहा छैन |
| 11 | खकार | हो | हैन | थाहा छैन |
| 12 | सास फेर्न गार्हो हुनु | हो | हैन | थाहा छैन |
| 13 | छाती घ्यार घ्यार हुने | हो | हैन | थाहा छैन |
| 14 | कुनै लक्षण थाहा नभएको | हो | हैन | थाहा छैन |
| **दिर्घ दम जोखिम तत्व बारे** | |  |  |  |
| 15 | धुम्रपान सेवन गर्नाले | हो | हैन | थाहा छैन |
| 16 | नियामित कोइला, दाउरा तथा गुइठाबाट आउने धुवा | हो | हैन | थाहा छैन |
| 17 | वायु प्रदूषण | हो | हैन | थाहा छैन |
| 18 | रक्सी सेवन | हो | हैन | थाहा छैन |
| 19 | कुनै जोखिम तत्व बारे थाहा नभएको | हो | हैन | थाहा छैन |
| **धुम्रपान र कोइला, दाउरा तथा गुइठाबाट आउने धुवा बारे** | |  |  |  |
| 20 | चुरोट खाने वा धुम्रपान त्याग्नाले दिर्घ दम रोग का लक्षण र समस्याहरु मा सुधार ल्याउन मद्घत गर्छ । | हो | हैन | थाहा छैन |
| 21 | नियामित कोइला, दाउरा तथा गुइठाबाट आउने धुवा बाट टाढा बस्नाले दिर्घ दम रोग लाई थप खराब हुन बाट रोक्न सकिन्छ । | हो | हैन | थाहा छैन |
| 22 | चुरोट वा धुम्रपान बाट निस्किने धुवाँ नै दिर्घ दम रोग को मुख्य कारण हो । | हो | हैन | थाहा छैन |
| **दिर्घ दम रोग को औषधी र उपचार बारे** | |  |  |  |
| 23 | दिर्घ दम रोग भएका मानिसहरूलाई इन्फ्लुएन्जा र निमोनिया विरुद्ध खोप लगाउनु पर्छ? | हो | हैन | थाहा छैन |
| 24 | दिर्घ दम रोगी ले इन्हेलर प्रयोगले रोगलाई अझ खराब हुनबाट रोक्छ? | हो | हैन | थाहा छैन |
| 25 | दिर्घ दम रोग लगेपछी गर्ने सास फेर्ने तरिका बाट गरिने फोक्सो को ब्यायाम र अरु सारिरिक व्यायामा बारे थाहा छ? | हो | हैन | थाहा छैन |
| 26 | दिर्घ दम r दैनिक रुपमा शारीरिक अभ्यास गर्ने र सकृय रहनाले फोक्सोको स्वास्थ्य सुधार गर्न मद्दत गर्छ? | हो | हैन | थाहा छैन |
