# Supplementary material for: Community-based management of chronic obstructive pulmonary disease in Nepal—Designing and implementing a training program for Female Community Health Volunteers
Source: PLOS Glob Public Health. 2022 Mar 25;2(3):e0000253. doi: 10.1371/journal.pgph.0000253 (PMC10021247; doi:10.1371/journal.pgph.0000253)
Supplement: S4 Appendix — (DOCX) [file pgph.0000253.s005.docx]

**S4_Appendix:** COBIN-P FCHVs Knowledge and Skills Evaluation/Supervision Form

**FCHVs Name:**

**Ward No:**

| **S.N.** | **Knowledge** | **Yes** | **No** | **Remarks** |
| --- | --- | --- | --- | --- |
|  | COPD |  |  |  |
|  | Risk factors |  |  |  |
|  | Sign and symptoms |  |  |  |
|  | Ways of prevention and control |  |  |  |
|  | Ways of diagnosis of COPD |  |  |  |
|  | Eligible age group |  |  |  |

| **S.N.** | **Skills** | **Yes** | **No** | **Remarks** |
| --- | --- | --- | --- | --- |
|  | Correct recording in Register |  |  |  |
|  | Use of CAF guide and counselling |  |  |  |
|  | Correct message through flip chart |  |  |  |
|  | Correct message through pamphlet etc. |  |  |  |
|  | Skills on breathing and stamina building exercise |  |  |  |

| **S.N.** | **Commodities** | **Yes** | **No** | **Remarks** |
| --- | --- | --- | --- | --- |
|  | Register |  |  |  |
|  | Pamphlet |  |  |  |
|  | Referral card |  |  |  |
|  | CAF guide |  |  |  |
|  | Exercise chart |  |  |  |

| **Overall Impression of FCHVs activities** | **Excellent** | **Good** | **Poor** |
| --- | --- | --- | --- |
|  |  |  |  |

Supervisor/Evaluator Name:

Signature:

Date:
