# Supplementary material for: Community-based management of chronic obstructive pulmonary disease in Nepal—Designing and implementing a training program for Female Community Health Volunteers
Source: PLOS Glob Public Health. 2022 Mar 25;2(3):e0000253. doi: 10.1371/journal.pgph.0000253 (PMC10021247; doi:10.1371/journal.pgph.0000253)
Supplement: S5 Appendix — (DOCX) [file pgph.0000253.s006.docx]

**S5_Appendix: Topic guide for COBIN-P Key Informant In-depth Interview for female community health volunteers, and Health Workers in English**

**Discussion Topic Guide for FCHVs**

- - Knowledge/awareness on COPD (risk factors, symptoms, treatment, management)?
  - Healthcare service points for COPD/CRDs treatment?
  - What are Religious and cultural perspective to chronic respiratory disease (Name, other practises in Chronic respiratory disease)?
  - What is Home based/local management of chronic respiratory disease (COPD)
  - How is the health care seeking practice in COPD?
  - Where do people go for chronic respiratory disease/COPD.
  - What is their chief complaint for respiratory related disease?
  - Who seeks care/make decision if a person becomes CRD related illness? Male? Female? Head of the family?
  - Do they approach you with CRDs/COPD?
  - What do you do in such situation?
  - At what stage or what kind of chief complain they come up with
  - What do you think major barriers of COPD disease treatment and management in our community?
  - What do you think major barriers of COPD disease treatment and management in our health system?
  - What are the prevailing facilitators/supporting factors of COPD treatment?
  - What kind of intervention is possible from FCHVs level?
  - What kind of interventions? List them
- **Additional questions for FCHVs in training/ intervention arm**
  - Satisfactory points of COBIN P training.
  - Unsatisfactory points of COBINP training
  - Positive aspects of changes made by COBIN P training on FCHVS
  - What are the barriers and challenges of this intervention implementation?
  - Suggestions for improvements.

**Topic guide for discussion/ interview with healthcare workers**

- - What are religious, cultural and local practise and perspective to chronic respiratory disease (Name, other practises in Chronic respiratory disease)?
  - What are the chief complaints of people with CRDs pa COPD?
  - What are the treatments available locally?
  - How do they manage the patients with COPD?
  - Where do they refer?
  - What are the challenges of COPD prevention, treatment and management locally?
  - What kind of public health interventions can be done locally to combat COPD?
  - What roles could FHCVs play in COPD prevention, treatment and management?
  - What are the suggestions for COPD prevention treatment and management in local context?
