# Supplementary material for: Community-based management of chronic obstructive pulmonary disease in Nepal—Designing and implementing a training program for Female Community Health Volunteers
Source: PLOS Glob Public Health. 2022 Mar 25;2(3):e0000253. doi: 10.1371/journal.pgph.0000253 (PMC10021247; doi:10.1371/journal.pgph.0000253)
Supplement: S6 Appendix — (DOCX) [file pgph.0000253.s007.docx]

**S6 Appendix: Topic guide for COBIN-P Key Informant In-depth Interview for female community health volunteers, and Health Workers in Nepali language**

**कोबिन पि: महिला स्वास्थ्य स्वयंम सेविका र स्वास्थ्यकर्मी अन्तर्वाता/छलफलका लागि आधार प्रश्नहरु**

महिला स्वास्थ्य स्वयंम सेविका अन्तर्वाता/छलफल का लागि आधार प्रश्नहरु ।

- दिर्घ श्वास प्रश्वास सम्बन्धी रोग (सिआरडी) र बिशेषत दिर्घ दम रोग/दम खोकी (सिओपिडी) जोखिम कारक, लक्षण, उपचार, व्यवस्थापन) बारे ज्ञान चेतना जानकारी ।
- (सिआरडी/सिओपिडी) उपचारको लागि स्वास्थ्य सेवाको उपलब्धता र उपचार सुबिधा बारे ।
- (सिआरडी/सिओपिडी)रोगको कारक र उपचार का लागि स्थानिय, धार्मिक र सांस्कृतिक अभ्यास के कस्तो छ (सिओपिडीका अन्य नामहरु, अन्य अभ्यासहरू) ।
- सिओपिडीको घर आधारित/स्थानीय व्यवस्थापन के कसरी हुने गरेको छ ।
- सिओपिडी समस्या हुँदा स्वास्थ्य उपचारका लागि स्वास्थ्य संस्था जाने अभ्यास कस्तो छ ।
- सिओपिडीको उपचारका लागि मानिसहरू कहाँ जाने गरेका छन ।
- श्वासप्रश्वाससम्बन्धी रोगको लागि उनीहरूको मुख्य गुनासो वा समस्या के हुने गरेको बताउने गरेका छन ।
- यदि कुनै व्यक्ति सिओपिडी सम्बन्धित बिरामी भएमा हेरचाह/निर्णय कसले खोज्छ पुरुष महिला परिवारको मुखिय मान्छे ।
- के तिनीहरूले तपाईंलाई सिओपिडीको समस्या लिएर सम्पर्क गर्ने गरेका छन ।
- यस्तो अवस्थामा के गर्नुहुन्छ ।
- मानिसहरू रोग को कुन कुन चरणमा वा कस्तो प्रमुख गुनासो लिएर आउँछन् ।
- हाम्रो समुदायमा सिओपिडी रोगको उपचार र व्यवस्थापनका प्रमुख बाधाहरू के के छन् जस्तो तपाईंलाई लाग्छ ।
- हाम्रो स्वास्थ्य प्रणालीमा सिओपिडी रोगको उपचार र व्यवस्थापनका प्रमुख बाधाहरू के-के लाग्छन् ।
- सिओपिडी उपचारका प्रचलित सहजकर्ता/सहायक कुराहरु के छन ।
- महिला स्वास्थ्य स्वयंम सेविकाको स्तरबाट सिओपिडी रोकथाम र नियन्त्रणमा कस्तो प्रकारको कामहरु सम्भव छन ।
- कस्तो प्रकारको कामहरु । जस्तै तिनीहरूलाई सूचीबद्ध गर्नुहोस् ।

कोबिण पि को तालिम लिएका F महिला स्वास्थ्य स्वयंम सेविकाकोहरूका लागि थप प्रश्नहरू

- कोबिण पि को तालिम प्रतिको सन्तुष्टि असन्तुस्टी बारे ।
- प्रशिक्षण सकारात्मक पक्षहरू ।
- प्रशिक्षण कमजोर पक्षहरू ।
- समुदायमा हाम्रो कार्यक्रम प्रती वा तपाईं हरुको यस् काम प्रती को धारणा ।
- तालिममा सिकेका ज्ञान वा जानकारी कार्यान्वयनका बाधा र चुनौतीहरू के हुन् ।
- सुधारको लागि कुनै सुझाव छ । तिनीहरूलाई सूचीबद्ध गर्नुहोस् ।

**मेडिकल अफिसर र स्वास्थ्य सहायकको अन्तर्वाता/छलफल का लागि आधार प्रश्नहरु**

- सिआरडी/सिओपिडी नाम, पुरानो श्वासप्रश्वास रोगमा अन्य अभ्यासहरू को धार्मिक, सांस्कृतिक र स्थानीय अभ्यास के कस्तो रहेको छ ।
- सिओपिडी भएका व्यक्तिहरूको मुख्य कस्तो कस्तो समस्या वा गुनासोहरू लिएर आउछन ।
- सिओपिडी का लागि स्थानीय रूपमा उपलब्ध उपचारहरू के के छन् ।
- उनीहरूले सिओपिडी भएका बिरामीहरूलाई कसरी व्यवस्थापन गर्छन् ।
- तिनीहरू कहाँ रिफर हुन्छन ।
- स्थानीय रूपमा सिओपिडी रोकथाम, उपचार र व्यवस्थापनका चुनौतीहरू के छन् ।
- सिओपिडी रोक्थाम र नियन्त्रन का लागि स्थानीय रूपमा के कस्तो काम हरु गर्न सकिन्छ ।
- सिओपिडी रोकथाम, उपचार र व्यवस्थापनमा महिला स्वास्थ्य स्वयंम सेविका हरुले ले के भूमिका खेल्न सक्छन ।
- स्थानीय सन्दर्भमा सिओपिडी रोकथाम उपचार र व्यवस्थापनको लागि सुझावहरू के छन् ।
